# Sedentary behaviour and disease risk

**DOI:** 10.1186/s12889-023-16867-2

**Published:** 2023-10-19

**Authors:** Joseph Henson, Marieke De Craemer, Thomas Yates

**Affiliations:** 1 NIHR Leicester Biomedical Research Centre, Diabetes Research Centre, College of Life Sciences, University of Leicester, LE5 4PW Leicester, UK; 2 Department of Rehabilitation Sciences, Ghent University, Ghent, Belgium

## Abstract

Sedentary behaviour has become the new reference of living, which has paralleled the increase in the prevalence of multiple chronic diseases. Here, we highlight the evidence to date and propose specific topics of interest for the Collection at BMC Public Health, titled “Sedentary behaviour and disease risk”.

## Main text

In modern society, sedentary behaviour has become the new reference of living. Sedentary behaviour, defined as waking time spent sitting or lying with low energy expenditure (≤ 1.5 metabolic equivalents) [1], occupies a large proportion of waking hours (up to 80%) across multi-faceted environments (e.g., home, work, school, transport). Given the complexity of quantifying sedentary behaviour, particularly in a free-living environment, it is typically operationalised as total daily sitting time, time spent watching television, or low levels of movement below a threshold on a wearable device. The definition of sedentary behaviour is fundamentally different to that of physical inactivity; the latter of which is most commonly used to categorise those not achieving the minimum recommendations of moderate-to-vigorous intensity physical activity (MVPA) (i.e., 150–300 min per week) [2]. Thus, it is possible for someone not to take part in any formal MVPA, yet engage in very little sedentary behaviour. Conversely, an individual who complies with current physical activity recommendations may still be highly sedentary. This distinction is at the heart of the sedentary behaviour paradigm.

The preference for engaging in sedentary behaviour has paralleled the increase in the prevalence of multiple chronic diseases (e.g. type 2 diabetes (T2DM), cardiovascular disease (CVD) and obesity) and their associated complications (e.g., sarcopenia, impaired physical function and frailty). As these non-communicable diseases account for 74% of all global deaths, of which 40% occur before the age of 70 [3], reiterating the importance of modifiable risk behaviours (e.g., sedentary behaviour) for the prevention of morbidity and premature mortality is an ongoing priority.

Compared to physical activity, which has been at the forefront of health research for decades, the literature on sedentary behaviour is in its infancy, with much of the evidence on the detrimental health effects associated with sedentary behaviour accumulated within the past decade. That said, recent updates to global physical activity guidelines now include the importance of reducing sedentary behaviour, providing a broad (‘limit the amount of time spent being sedentary and replace with physical activity of any intensity’) set of behavioural targets that complement the well-established recommendations for physical activity [2], thus marking an important step forward. Although the level of evidence was insufficient to promulgate a specific threshold, these broad guidelines encompass the burgeoning epidemiological evidence examining the association between greater time spent in sedentary behaviour (examined mostly via self-report or device-based assessments of sitting or television viewing time) and higher all-cause and cardiovascular mortality, alongside the higher incidence of CVD and T2DM [4, 5]. For example, the dose-response relationship between sedentary time and all-cause mortality appears to increase gradually from ~ 7.5 h/day and is more pronounced at > 9.5 h/day [4]. More specifically, sitting for 10 h/day is associated with a 48% increased risk of all cause-mortality (vs. 7.5 h/day) [4].

When examining the relationships between sedentary behaviour and markers of health, it is also important to consider the interplay with other 24-hour physical behaviours i.e., each outcome should not necessarily be considered in isolation. In this context, a 24-hour day comprises a sequence of movement behaviours distributed on a continuum ranging from limited/no movement to high-intensity activities. For instance, in those individuals who engage in 30–40 min of MVPA per day, the association between high sedentary time (> 10.7 h/day) and risk of death is not meaningfully different from those with low amounts sedentary time (< 8.5 h/day) [6].

Over recent years, epidemiological research has been complemented by acute experimental studies showing that breaking up bouts of prolonged sitting with standing or light-intensity activity (including resistance exercises) elicits significant benefits on markers of cardiometabolic health [7–9]. However, the primary focus of chronic sedentary behaviour studies so far has been on the behavioural efficacy of the proposed interventions, therefore, the promising results have yet to be replicated in chronic, free-living interventions.

Indeed, within sedentary behaviour intervention research, and public health campaigns more broadly, there is a substantial lack of evidence for the long-term health benefit and major barriers to translation exist (i.e., evidence to practice). As such, community or “systems-based” approaches are needed to address the multiple determinants of sedentary behaviour that focus on combining both upstream policy approaches (i.e., aimed at improving the social, cultural, economic and environmental factors that support reductions in sedentary behaviour) and downstream individual-focused (i.e., education and information) strategies.

As previously mentioned, people engage in sedentary behaviour in different contexts and the built environment can play an important role in influencing sedentary behaviour. Therefore, the need to develop and evaluate context and behaviour-specific, multicomponent, complex interventions that incorporate environmental modifications (e.g., outdoor spaces or work) to reduce sedentary behaviour are essential. For example, the SMART WORK and Life study demonstrated that when a sedentary behaviour intervention conducted in a workplace environment (and delivered by “workplace champions”) was coupled with a height adjustable desk, it resulted in a > 60min reduction in sitting time (vs. control) [10].

This Collection at BMC Public Health, “Sedentary behaviour and disease risk”, offers an exciting opportunity to augment the aforementioned research evidence by publishing high-quality, multi-disciplinary research relating to sedentary behaviour and health (across single or multiple chronic diseases). We are also interested in public awareness campaigns, policies and context specific interventions for sedentary behaviour. As considerable inter-individual variability exists, we also embrace research exploring the modification effect of key demographic, social, cardiometabolic and anthropometric characteristics.

Word count: 877.

## Data Availability

Not applicable.

## List of Abbreviations

CVD: Cardiovascular disease
MVPA: Moderate–to–vigorous physical activity
T2DM: Type 2 diabetes mellitus
